# Therapeutic Potential of Phellodendrine/ Atractylenolide-I Combination in Rheumatoid Arthritis: Targeting Neutrophil Apoptosis through LTB4/BLT1 Pathway Suppression

**DOI:** 10.2174/0118715303404238251210141302

**Published:** 2026-05-21

**Authors:** Zhifang Qin, Ran Tang, Gege Jiang, Jiahua Yin, Shili Xu, Qi Wang, Kaiyin Cai, Juan Liang, Min Zhang, Xiaoyi Jia

**Affiliations:** 1 School of Pharmacy, Anhui University of Chinese Medicine, Hefei, 230012, China;; 2 Department of Rheumatology and Immunology, The First Affiliated Hospital of USTC, Division of Life Sciences and Medicine, University of Science and Technology of China, Hefei 230001, China

**Keywords:** Phellodendron, atractylodendron-I, neutrophil, apoptosis, LTB4/BLT1 pathway, rheumatoid arthritis

## Abstract

**Introduction:**

To investigate the therapeutic effects of rheumatoid arthritis (RA) Phellodendrine and Atractylenolide-I (PHE/ATL-1) on RA and their mechanisms.

**Methods:**

In this study, first clinical experiments were conducted to investigate whether the delayed neutrophil apoptosis in the course of RA was related to the Leukotriene B4 (LTB4)/ Leukotriene B4 receptor 1 (BLT1) pathway, and then the effects of PHE/ATL-1 inhibition of the LTB4/BLT1 pathway to promote neutrophil apoptosis to inhibit inflammation were evaluated *in vitro,* as well as the *in vivo* effects of PHE/ATL-1 on the collagen-induced arthritis (CIA) model.

**Results:**

Clinical studies revealed elevated BLT1 expression in RA neutrophils, impairing apoptosis and exacerbating inflammation. *In vitro,* PHE/ATL-1 restored neutrophil apoptosis and reduced inflammation by inhibiting the LTB4/BLT1 pathway. The CIA mouse model further confirmed PHE/ATL-1's therapeutic potential in alleviating arthritis symptoms.

**Discussion:**

PHE/ATL-1 ameliorates RA through LTB4/BLT1 pathway inhibition, reversing neutrophil apoptosis delay and attenuating inflammation. Clinical evidence of upregulated BLT1 in RA neutrophils and therapeutic efficacy in both *in vitro* and CIA models establishes PHE/ATL-1 as a compelling RA treatment candidate requiring clinical validation.

**Conclusion:**

These findings suggest that PHE/ATL-1 may serve as a potential therapeutic agent for RA treatment.

## 1 INTRODUCTION

Rheumatoid arthritis (RA) is a chronic systemic autoimmune disease [1]. Its pathogenesis primarily manifests in the synovium of the joints, which remains in a state of long-term inflammation, leading to abnormal proliferation of the synovium and eventual cartilage damage [2]. If not effectively controlled over time, RA may result in varying degrees of disability. The pathogenesis of RA is not yet fully understood. Previous studies have suggested that RA is related to disorders of specific immunity, such as dysfunctions of T- cells and B-cells, while recent studies have shown that it is also closely associated with intrinsic immune disorders. In particular, the role of neutrophils in the pathogenesis and progression of RA has attracted attention [3]. Drugs currently widely used for the treatment of RA, such as methotrexate, leflunomide, tofacitinib, and glucocorticoids, can effectively modulate neutrophil function through various mechanisms [4]. Targeting neutrophil-associated cytokines and chemokine formation represents a potential therapeutic strategy, and further understanding of how neutrophils regulate autoimmunity and tissue damage in RA may facilitate the development of novel and effective therapies.

Neutrophils are the most numerous leukocytes in the blood and are produced daily in large numbers by the bone marrow [5, 6], accounting for 50% to 70% of total peripheral blood leukocytes. Studies have shown that blood neutrophils in RA exhibit an aberrant activation phenotype, characterized by increased reactive oxygen species, elevated cytokine production, and delayed apoptosis [7]. One important mechanism in RA pathogenesis involves the unbalanced regulation of neutrophil apoptosis, which can lead to either abnormal expansion or excessive cell death. Therefore, elucidating the relationship between neutrophil apoptosis and RA is crucial [8].

Leukotriene B4 (LTB4), a pro-inflammatory lipid mediator, is produced from arachidonic acid *via* 5-lipoxygenase, 5-lipoxygenase-activating protein, and LTA4 hydrolase [9]. LTB4 is rapidly produced and released by neutrophils, macrophages, and mast cells, and it plays a key role in the inflammatory response [10]. Leukotriene B4 receptor 1 (BLT1) is highly expressed on neutrophils. LTB4 binds to BLT1 with high specificity and affinity, inducing neutrophil chemotaxis and adhesion, and regulating LTB4 activity in neutrophils [11]. Several studies have demonstrated the critical role of LTB4 and BLT1 in the pathogenesis of inflammatory arthritis in mouse models [12]. It has also been shown that LTB4 and its receptor BLT1 exacerbate synovial inflammation *in vivo* (Critical Role of LTB4/BLT1 in IL-23-Induced Synovial Inflammation and Osteoclastogenesis *via* NF-kB). While the role of the LTB4/BLT1 axis in neutrophil apoptosis has not yet been reported in RA, targeting this pathway may provide a new strategy for RA treatment.

Natural herbs have been used to treat autoimmune diseases, including RA. The group's preliminary study found that Ermiao San has therapeutic effects on RA and identified Phellodendrine (PHE) and Atractylenolide-I (ATL-1) as key components of Phellodendron amurense [13, 14]. PHE is a natural isoquinoline alkaloid derived from various Chinese medicinal herbs, primarily found in Phellodendron amurense [15]. As the main active ingredient of Phellodendron amurense, its alkaloids are widely used in traditional Chinese medicine to clear heat, dry dampness, reduce diarrhea, and detoxify toxins [16]. In recent years, with the development of modern pharmacology, the diverse biological activities of Phellodendron alkaloids have been gradually revealed, demonstrating their potential in treating various inflammatory diseases [17].

ATL-1 is a sesquiterpene lactone extracted from the traditional Chinese medicine Atractylodes macrocephala, which exhibits significant biological activities. It has received widespread attention for its anti-inflammatory, anticancer, immunomodulatory, and neuroprotective effects, showing particular promise in the treatment of inflammatory and immune-related diseases [18, 19]. Although PHE and ATL-1 individually demonstrate therapeutic effects against inflammatory diseases, the mechanism of their combined action in RA remains unclear. Therefore, it is of great interest to investigate the combination of PHE and ATL-1 in RA. The present study focuses on the LTB4/BLT1 pathway-mediated neutrophil apoptosis as a target to determine whether PHE/ATL-1 inhibits this pathway and promotes neutrophil apoptosis to treat RA.

## 2 MATERIALS AND METHODS

### 2.1 Experimental Materials

Blood samples were obtained from 30 RA patients (Table **1**) and 10 healthy controls (HC), and all RA patients met the criteria revised by the United States College of Rheumatology in 1987. The research protocol was approved by the Medical Ethics Committee of the First Affiliated Hospital of the University of Science and Technology of China (protocol number: 2022-KY154).

#### 2.1.1 Experimental Animals (CIA Model)

- Species and strain: DBA/1 mice
- Gender: Male
- Age: 8 weeks old
- Weight: 20-22 g (at the time of purchase)
- Source: Purchased from SLAC Laboratory Animal Centre (SCXK (Shanghai) 2017-0005)
- Breeding conditions: Raised in the Laboratory Animal Centre of Anhui University of Traditional Chinese Medicine (AHUCM) under controlled environment (temperature 22-26°C, light-dark cycle 12 h/12 h)
- Sample size: 35 mice in total, 7 mice per group
- Grouping: Randomly divided into normal group, model group, low-dose PHE/ATL-1 group (150:75 mg/kg), high-dose PHE/ATL-1 group (75:37.5 mg/kg), and methotrexate (MTX) group

#### 2.1.2 Experimental Animals (Neutrophil Inflammation Model)

- Species and strain: Kunming mice
- Gender: Male
- Age: 8 weeks old
- Weight: 20-24 g (at the time of purchase)
- Source: Purchased from SLAC Laboratory Animal Centre (SCXK (Shanghai) 2017-0005)
- Breeding conditions: Raised in the Laboratory Animal Centre of Anhui University of Traditional Chinese Medicine (AHUCM) under controlled environment (temperature 22-26°C, light-dark cycle 12 h/12 h)
- Sample size: 24 mice in total, 6 mice per group; an additional 50 Kunming mice were used for neutrophil extraction in cellular experiments
- Grouping: Randomly divided into normal group, lipopolysaccharide (LPS) group, LPS+PHE/ATL-1 group, and LPS+PHE/ATL-1+LTB4 group

The animal experimentation was approved by the Ethics Committee of Anhui University of Traditional Chinese Medicine (Ethics No. AHUCM-mouse-2023134).

### 2.2 Drugs and Chemicals

PHE (Cat. CFN99944) and ATL-1 (Cat. CFN99143) were obtained from Chem Faces (Wuhan, China); BCG vaccine was purchased from Beijing Biological Products Research Institute Co. (Beijing, China); chicken type II collagen was obtained from Chondrex (Washington DC, USA); LTB4 agonist (HY-107608, MCE); liquid paraffin was obtained from Shanghai Yuanye Biotechnology Co. Kit (EK206/3; Multi Sciences); Mouse IL-6 Enzyme-Linked Immunosorbent Assay Kit (ml098430; MEIMIAN); Mouse LTB4 Enzyme-Linked Immunosorbent Assay Kit (ml201802; MEIMIAN;); Mouse TNF-α Enzyme-Linked Immunosorbent Assay Kit (ml002095; MEIMIAN); Mouse IL-Iβ Enzyme-Linked Immunosorbent Assay Kit (YJ201802; MEIMIAN);CCK8 Detection Kit (BS350B; biosharp); Human Peripheral Blood Neutrophil Kit (P9040; Solarbio); Annexin V-FITC/PI Double Staining Apoptosis Detection Kit (BB23061; Bestbio); Anti-Bcl-2 Antibody (ET1702- 53; HUABIO), anti-Bax antibody (ET1742-53/ET1603-34; HUABIO); anti-GAPDH antibody (AF7021; Affinity); secondary rabbit antibody (SA00001-1; Proteintech); BLT1 primer (2512710559-60;Shengong Bioengineering); GAPDH primer (H510011-0002;Shengong Bioengineering).

### 2.3 CIA Induction and Evaluation

Chicken type II collagen solution was mixed with CFA in equal amounts and ground repeatedly until completely emulsified to form a 1 mg/mL emulsion. DBA/1 mice were injected intradermally with 0.15 mL of the emulsion at the root of the tail on day 0 (primary immunisation) to induce inflammation, followed by a second injection of 0.1 mL of the emulsion at the same site on day 21 (secondary immunisation). The normal group was injected with saline in the same manner.

After administration of the modelling agent, the mice were divided into the normal group, the model group, the PHE/ATL-1 low-dose group, the PHE/ATL-1 high-dose group, and the methotrexate (MTX) group. PHE and ATL-1 were administered orally once daily starting on day 28, while the positive control MTX group received gavage once every three days for four weeks. Mice were sacrificed on day 60 for observation of relevant parameters. The diameter of the ankle joints was measured using a pocket thickness gauge (Mitutoyo, Kawasaki, Japan). The severity of arthritis was scored from 0 to 4, with a maximum achievable score of 4 per mouse. Both arthritis scores and ankle joint diameters were recorded every three days during the treatment period.

### 2.4 Sample Preparation

After the mice were anaesthetised, blood was collected from the eye canthus. The blood was centrifuged at 3000 rpm for 10 min, and the serum was stored at -80°C for further use. Ankle joint specimens were collected immediately after euthanasia and fixed in 4% paraformaldehyde solution. Simultaneously, the thymus and spleen were removed in the cell room for processing.

### 2.5 Histopathology and Immunohistochemistry

The ankle joints of mice were decalcified and fixed in 10% EDTA solution and embedded in paraffin. Sections of 5 μm thickness were stained with hematoxylin-eosin (H&E) or safflower O-fast green following standard procedures. For immunohistochemistry, sections underwent microwave antigen retrieval in citrate buffer (pH 6.0), were quenched with hydrogen peroxide, and blocked with 3% bovine serum albumin at room temperature. Tissues were incubated overnight at 4°C with anti-MPO or anti-NE antibodies, followed by incubation with secondary antibodies for 30 min at 37°C. Slides were visualized using 3,3′-diaminobenzidine (DAB).

### 2.6 CCK-8 Experiments

Cell Counting Kit-8 (CCK-8) assays were used to assess the effect of different ratios of PHE/ATL-1 on neutrophil proliferation. Neutrophils were seeded in a 96-well plate and cultured until they reached approximately 80% confluence, after which the drugs were added. Experiments were performed according to the instructions provided with the CCK-8 kit (Biosharp, China).

### 2.7 Isolation and Culture of Peripheral Blood Neutrophils

#### 2.7.1 Isolation and Culture of Peripheral Blood Neutrophils

Peripheral blood was collected from the eye canthus of mice. Neutrophils were isolated using a mouse peripheral blood neutrophil isolation kit (Solebo). In a centrifuge tube, 4 mL of Reagent A was added first, followed by careful layering of 2 mL of Reagent C on top. Blood was then gently layered above the isolation solution, maintaining a clear interface between the two liquids. After centrifugation, two milky white layers appeared: the upper layer consisted of mononuclear cells, and the lower layer contained neutrophils. Neutrophils were carefully collected from the interface between Reagent C and Reagent A and transferred into a clean 15 mL centrifuge tube. Cells were washed with 10 mL of phosphate-buffered saline (PBS) or Cell Wash Solution and centrifuged at 250 × g for 10 min. The supernatant was discarded, and the cells were resuspended in 5 mL of PBS or Cell Wash Solution. This washing and centrifugation step was repeated once before final resuspension of the cells.

### 2.8 Flow Cytometry Analysis

Neutrophils were placed in EP tubes at a concentration of 1 × 10^6 cells/mL. After incubation, cells were washed twice with cold PBS by centrifugation at 400 × g for 5 min and then resuspended in 200 μL of 1× binding buffer. Five microliters of fluorescein isothiocyanate (FITC)-conjugated Annexin V were added, and cells were incubated in the dark for 15 min. Subsequently, 5 μL of propidium iodide (PI) was added, followed by a 5 min incubation at room temperature protected from light. Apoptosis was analysed by flow cytometry within 1 h.

### 2.9 Micro-computed Tomography (Micro-CT) Analysis

The right ankle joint was fixed in 10% formalin for 48 h, washed in PBS for 2 h, then immersed in 75% ethanol and scanned using a Micro-CT system (Scanco VIVA CT80, Scanco Medical AG, Switzerland). The scanning parameters were: pixel size 15.6 µm, tube voltage 55 kV, tube current 72 µA, and integration time 200 ms. 3D reconstruction and realignment of the cross-sectional images were performed using µCT Evaluation Program V6.6 (Scanco Medical AG, Switzerland). Bone volume was measured, with a density threshold set at 370-1000 for bone. A total of 340-441 cross-sections were reconstructed with an interlayer distance of 1 pixel (15.6 µm), corresponding to a reconstruction height of 5.3-6.9 mm, and the ankle joint was reconstructed.

### 2.10 ELISA for Inflammatory Factor Levels

Neutrophils obtained from isolation were suspended in RPMI 1640 containing 10% fetal bovine serum and inoculated into culture plates. The supernatant was collected, centrifuged for 5 min, and carefully removed for storage. ELISA kits were used to detect the expression levels of relevant inflammatory factors.

### 2.11 Extraction of Neutrophils For Cellular Experiments

Neutrophils were extracted from 50 Kunming mice *via* blood sampling from the eye canthus for subsequent cellular experiments. Kunming mice were purchased from SLAC Laboratory Animal Centre (SCXK [Shanghai] 2017-0005) and maintained in the Laboratory Animal Centre of Anhui University of Traditional Chinese Medicine under controlled conditions (22-26°C, 12 h light/12 h dark cycle).

### 2.12 Cellular Experiment Administration

Neutrophils were divided into the following groups: normal group, LPS group, LPS + PHE/ATL-1 group, and LPS + PHE/ATL-1 + LTB4 agonist group. Cells were seeded in 6-well plates at a density of 2 × 10^6 cells per well and cultured in a 37°C, 5% CO_2_ incubator for 24 h. Neutrophils were stimulated with LPS (100 ng/mL) for 4 h to mimic inflammatory conditions *in vivo*. PHE/ATL-1 treatment was applied for 6 h, and the LTB4 agonist was added for 6 h.

### 2.13 Western Blot (WB) Analysis

Proteins were separated using 10% sodium dodecyl sulfate-polyacrylamide gel electrophoresis (SDS-PAGE) and transferred onto a nitrocellulose membrane. Membranes were blocked with 5% skimmed dry milk in TBST buffer for 2 h at room temperature. They were then incubated overnight at 4°C with primary antibodies. After three washes with TBST, membranes were incubated with horseradish peroxidase-conjugated secondary antibodies for 1 h at room temperature. Following three additional washes with TBST, protein detection was performed using an enhanced chemiluminescence (ECL) reagent, and bands were visualized with a chemiluminescence imaging system. Protein band intensities were quantified using ImageJ software.2.14 RNA isolation and q-PCR.

Total RNA was extracted from isolated neutrophils, and real-time quantitative RT-PCR (qRT-PCR) was performed using human gene-specific primers. The expression of BLT1 and GAPDH was detected using primers and probes obtained from Sangon Biotech.

### 2.14 Statistical Analysis

Data are presented as mean ± standard error of the mean (SEM). Joint diameters and arthritis scores were analysed using two-way analysis of variance (ANOVA). For Western blot and ELISA experiments, statistical differences were determined using one-way ANOVA. Apoptosis data were compared using one-way ANOVA followed by the least significant difference (LSD) post hoc test. A *p*-value of less than 0.05 was considered statistically significant.

## 3 RESULTS

### 3.1 Delayed Neutrophil Apoptosis in the RA Process is Associated with the LTB4/BLT1 Pathway

To investigate whether RA progression is associated with delayed neutrophil apoptosis mediated by the LTB4/BLT1 pathway, clinical samples were collected from 30 RA patients (Table **1**). ELISA results showed that LTB4 levels in peripheral blood neutrophils of RA patients were significantly higher compared to healthy controls (HC) (Fig. **1A**). Western blot and qPCR analyses confirmed elevated BLT1 protein and mRNA expression in peripheral blood neutrophils of RA patients (Figs. **1B**-**D**). Additionally, apoptosis in peripheral blood neutrophils from RA patients and healthy controls was assessed by flow cytometry. The results demonstrated that neutrophil apoptosis in RA patients was significantly reduced compared to healthy volunteers (Figs. **1E** and **F**). These findings suggest that delayed neutrophil apoptosis in RA is closely associated with activation of the LTB4/BLT1 pathway.

### 3.2 Cell Experiments Determined the Optimal PHE/ATL-1 Ratio as 2:1

To determine the optimal ratio of PHE/ATL-1, CCK-8 analysis was performed to assess the effects of different concentrations of PHE and ATL-1 on neutrophil activity, and appropriate concentrations were identified for subsequent experiments. Neutrophil activity was first measured using the CCK-8 assay at various concentrations of PHE and ATL-1. Based on these results, PHE concentrations of 200 μM and 100 μM, and an ATL-1 concentration of 100 μM were selected as the screening concentrations for further experiments (Figs. **2A** and **B**). Neutrophils were then treated with PHE/ATL-1 at ratios of 2:1 and 1:1 for 6 hours. The results indicated that different ratios of PHE and ATL-1 affected neutrophil activity, with the 2:1 ratio being optimal. Accordingly, a PHE concentration of 200 μM and an ATL-1 concentration of 100 μM at a 2:1 ratio (Fig. **2C**) was selected for subsequent cellular experiments.

### 3.3 PHE/ATL-1 Inhibits LTB4/BLT1 Pathway to Promote Neutrophil Apoptosis to Suppress Inflammation

To investigate whether PHE/ATL-1 promotes neutrophil apoptosis to inhibit inflammation *via* the LTB4/BLT1 pathway, apoptosis rates were first measured by flow cytometry. Neutrophils in the normal group exhibited higher apoptosis compared to the LPS group, indicating that the inflammatory model was successfully established. The LPS + PHE/ATL-1 group showed a significant increase in apoptosis relative to the LPS group, whereas the LPS + PHE/ATL-1 + LTB4 group displayed significantly lower apoptosis compared to the LPS + PHE/ATL-1 group, suggesting that the LTB4 agonist can reverse the pro-apoptotic effect of PHE/ATL-1 (Figs. **3A** and **B**).

Bax and Bcl-2 are key regulators of neutrophil apoptosis. To further investigate, neutrophils were stimulated with LPS + PHE/ATL-1, and Western blot analysis confirmed that PHE/ATL-1 promoted neutrophil apoptosis (Figs. **3C** and **D**). Changes in supernatant levels of inflammatory factors TNF-α, IL-1β, IL-6, and LTB4 were assessed by ELISA, showing that PHE/ATL-1 significantly reduced inflammation, while the LTB4 agonist reversed this effect (Fig. **3E**).

These results demonstrate that PHE/ATL-1 inhibits the LTB4/BLT1 pathway to promote neutrophil apoptosis and suppress inflammation.

### 3.4 PHE/ATL-1 Alleviates Arthritis Symptoms in CIA Mice

To investigate whether PHE/ATL-1 could reduce arthritis symptoms in CIA mice, we evaluated paw and foot swelling, arthritis scores, ankle diameter, and digit swelling rates following modelling and treatment. Additionally, we assessed blood flow signals, Micro-CT imaging, and histopathology of the knee joints using H&E staining. RA symptoms were evident 7 days after administration of the modelling agent in DBA/1 mice, while no signs of inflammation were observed in the normal group. Mice treated with the modelling agent showed significant increases in ankle joint diameter and arthritis scores, which were measured every 3 days during treatment.

Representative images demonstrated that paw swelling, arthritis scores, foot swelling, and digit swelling were significantly lower (*P* < 0.01) in PHE/ATL-1-treated and MTX-treated groups compared with the saline-treated model group (Figs. **4A**-**D**). Small animal ultrasound imaging revealed increased blood flow in the knee joints of the model group, which was reduced in the PHE/ATL-1-treated group (Fig. **5A**), indicating a therapeutic effect.

Histomorphometric Micro-CT analysis showed that the saline-treated model group exhibited extensive cartilage and bone damage, rough bone surfaces, narrowed joint spaces, severe joint erosion, and significantly reduced bone volume. PHE/ATL-1 treatment reduced the severity of joint erosion, with the high-dose group showing greater protection than the low-dose group (Fig. **5B**). H&E histopathology confirmed that PHE/ATL-1 inhibited joint erosion, reduced synovial proliferation, decreased inflammatory cell infiltration, and improved cartilage integrity in mouse ankle joints (Fig. **5C**).

These results collectively suggest that PHE/ATL-1 exerts a significant therapeutic effect in CIA mice.

### 3.5 PHE/ATL-1 Promotes Neutrophil Apoptosis and Inhibits Inflammation in Mice

To investigate whether PHE/ATL-1 promotes neutrophil apoptosis to inhibit inflammation in mice, we first assessed its effects on neutrophils isolated from DBA/1 mice using flow cytometry. Neutrophils were stained with Annexin V/PI prior to analysis. The results showed that neutrophil apoptosis (Q2 + Q4) was decreased in the model group, while PHE/ATL-1 treatment significantly increased apoptosis (Figs. **6A** and **B**).

Bax and Bcl-2 are key regulators of neutrophil apoptosis. Immunohistochemical analysis revealed that PHE/ATL-1 significantly increased the expression of the pro-apoptotic protein Bax and decreased the expression of the anti-apoptotic protein Bcl-2 in the model group (Fig. **6C**).

Serum was collected and analyzed by ELISA to evaluate the effects of PHE/ATL-1 on cytokine levels. Proinflammatory cytokines, including TNF-α, IL-1β, and IL-6, were consistently lower in the normal group compared with the saline-treated model group. PHE/ATL-1 treatment reduced the levels of TNF-α, IL-1β, and IL-6 compared with the model group. LTB4, a key mediator in the inflammatory pathway, was also reduced in the PHE/ATL-1-treated group compared with the model group (Fig. **6D**).

These results indicate that PHE/ATL-1 promotes neutrophil apoptosis to suppress inflammation in mice.

## 4 DISCUSSION

RA is a chronic, systemic autoimmune disease that primarily affects the synovium of the joints, resulting in pain, swelling, and impaired function. Its pathology is characterized by synovial inflammation and proliferation, which can ultimately lead to destruction of articular cartilage and bone, and in severe cases, joint deformity and disability [20, 21]. Although the exact etiology of RA is not fully understood, genetic, environmental, and immunological factors are known to contribute to its pathogenesis [22, 23].

Hyperactivation and delayed apoptosis of neutrophils are key mechanisms driving chronic inflammation in RA. Under normal conditions, neutrophils undergo rapid apoptosis after completing their immune functions, preventing excessive inflammation. In RA, however, neutrophil apoptosis is significantly delayed. This delay results in persistent inflammation, as neutrophils remain in the synovial fluid and continue to release pro-inflammatory factors such as TNF-α, IL-1β, and IL-6, exacerbating synovial inflammation. Additionally, neutrophils continuously release destructive molecules, including reactive oxygen species and proteolytic enzymes, which contribute to further cartilage and bone damage [24, 25].

Leukotriene B4 (LTB4) is a potent neutrophil chemotactic factor. By binding to its receptor BLT1, LTB4 induces neutrophil migration to inflammatory sites and activates them to release pro-inflammatory cytokines (*e.g.*, TNF-α, IL-1β) and reactive oxygen species, further amplifying the inflammatory response. In autoimmune diseases such as RA, aberrant activation of neutrophils *via* the LTB4/BLT1 pathway sustains synovial inflammation and joint damage [26]. LTB4 not only promotes neutrophil accumulation at the joint site but also delays their apoptosis, prolonging their participation in the inflammatory response and contributing to disease chronicity [27, 28]. Aberrant activation of the LTB4/BLT1 pathway is therefore considered a key mechanism underlying chronic inflammation and joint destruction in RA, as it drives both neutrophil accumulation in the joint cavity and overproduction of inflammatory mediators [29].

In this study, we found that the development of RA is closely associated with delayed neutrophil apoptosis, particularly through dysregulation of the LTB4/BLT1 pathway. We observed a significant increase in BLT1 protein and mRNA expression in neutrophils from RA patients, leading to impaired apoptosis and exacerbation of the inflammatory response. These findings provide new insights into RA pathogenesis and highlight the central role of the LTB4/BLT1 pathway.

*In vitro* experiments demonstrated that PHE/ATL-1, as a potential therapeutic agent, promotes neutrophil apoptosis and attenuates inflammation by inhibiting the LTB4/BLT1 pathway. Maintaining a balance between pro- and anti-inflammatory factors is critical in RA progression, and although our current data show effective suppression of inflammatory drivers, monitoring immunoregulatory cytokines in future clinical trials will further clarify therapeutic equilibrium. We further confirmed that PHE/ATL-1 effectively promotes mouse neutrophil apoptosis and inhibits the release of pro-inflammatory cytokines, as demonstrated by CCK-8, flow cytometry, Western blot, and ELISA analyses, highlighting its potential to modulate inflammatory responses in RA.

In a CIA mouse model, PHE/ATL-1 alleviated arthritis symptoms, reduced joint tissue damage, and improved immune function, supporting its promise as a therapeutic agent. Compared with traditional methotrexate (MTX), PHE/ATL-1 exhibits distinct pharmacological characteristics: MTX primarily inhibits dihydrofolate reductase, affecting purine metabolism, whereas PHE/ATL-1 specifically regulates neutrophil apoptosis mediated by the LTB4/BLT1 pathway. Our study also showed that PHE/ATL-1 not only promoted neutrophil apoptosis *via* regulation of Bax and Bcl-2 expression but also significantly reduced inflammatory cell infiltration and joint tissue damage in CIA mice.

These results underscore the potential of PHE/ATL-1 in RA therapy, particularly through regulation of neutrophil apoptosis to attenuate inflammation. Clinically, MTX requires 4-6 weeks to achieve therapeutic effects, whereas PHE/ATL-1 significantly improved joint swelling after just 7 days of treatment, demonstrating comparable anti-inflammatory efficacy. Moreover, while MTX primarily targets dihydrofolate reductase, PHE/ATL-1’s dual mechanism—targeting both neutrophil apoptosis and the LTB4/BLT1 pathway—may contribute to a superior safety profile, suggesting its potential as an alternative for patients intolerant to MTX.

In summary, the results of this study indicate that PHE/ATL-1 regulates neutrophil apoptosis by targeting the LTB4/BLT1 pathway, thereby alleviating inflammatory responses and providing new insights into the treatment of RA. However, further clinical studies are needed to verify the safety and efficacy of PHE/ATL-1 in the treatment of RA and to explore its optimal application strategy in RA treatment.

## CONCLUSION

Overall, this study provides a new perspective on the treatment of RA, namely, PHE/ATL-1 has the potential to become a new drug for RA therapy by targeting the LTB4/BLT1 pathway to regulate neutrophil apoptosis and inflammatory response. However, further clinical studies are still needed to validate its safety and efficacy and to explore its optimal application strategy in RA treatment.

## Figures and Tables

**Fig. (1):**
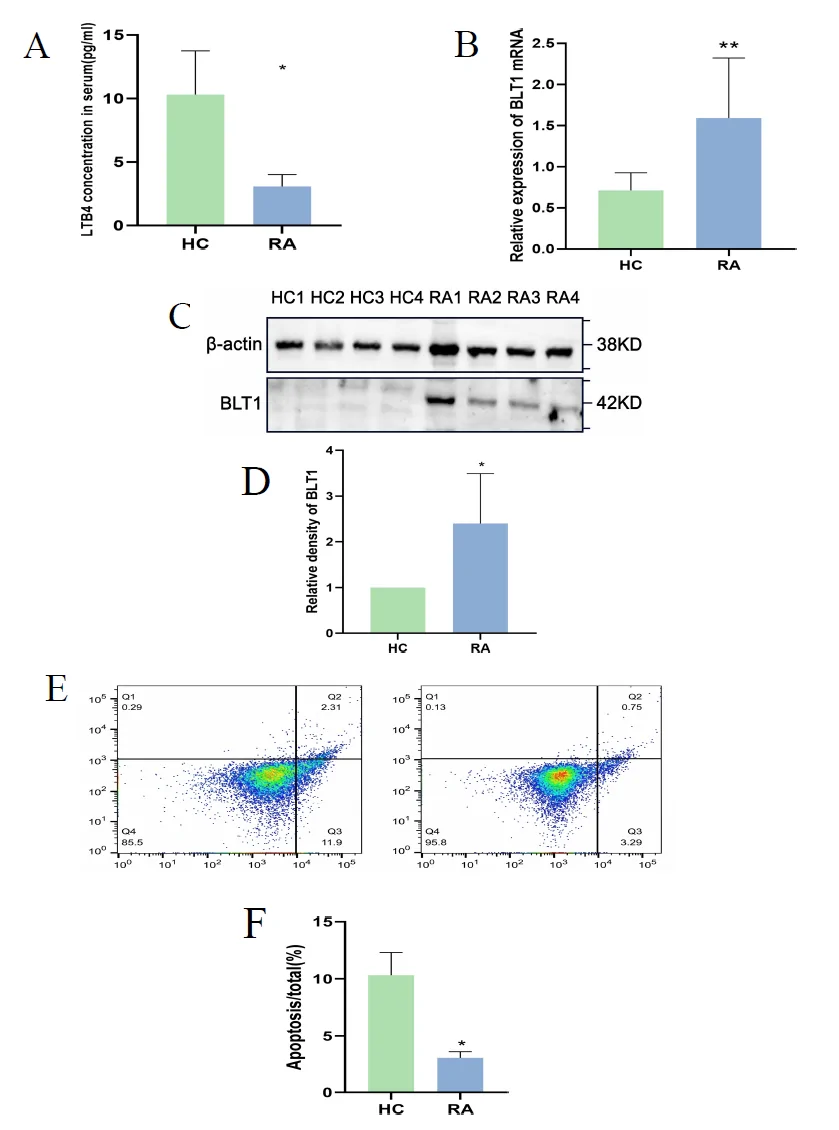
Delayed neutrophil apoptosis in RA is associated with the LTB4/BLT1 pathway. (**A**) ELISA detection of LTB4 levels in neutrophils. (**B**) PCR detection of BLT1 mRNA in neutrophils. (**C** and **D**) Western blot detection of BLT1 protein in neutrophils. (**E** and **F**) Flow cytometry detection of neutrophil apoptosis rate. **P<0.01, *P<0.05 compared with HC.

**Fig (2):**
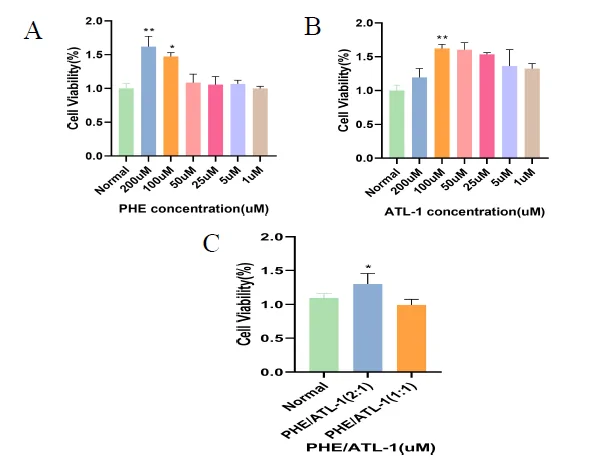
Investigation of the optimal ratio of PHE/ATL-1. (**A**) Effect of different concentrations of PHE on neutrophil activity; (**B**) Effect of different concentrations of ATL-1 on neutrophil activity; (**C**) Effect of different PHE/ATL-1 ratios on neutrophil activity (compared with the Normal group, *P<0.05, **P<0.01).

**Fig. (3):**
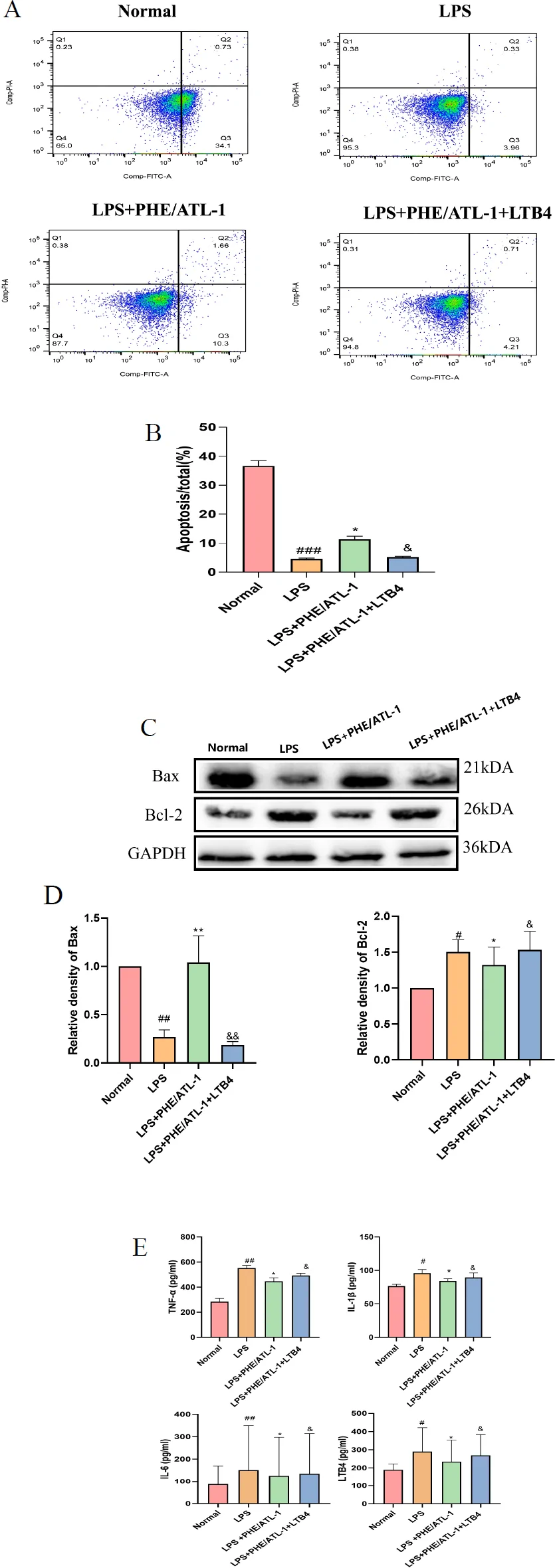
*In vitro* PHE/ATL-1 inhibits the LTB4/BLT1 pathway and promotes neutrophil apoptosis. (**A** and **B**) Flow cytometry was used to detect the rate of apoptosis. (**C** and **D**) Western blot detects apoptotic proteins. (**E**) Expression levels of TNF-α, IL-6,IL-1β, LTB4 in the serum of neutrophils . *P<0.05 , **P<0.01 *vs* LPS group ; ^#^*P*<0.05, ^##^*P*<0.01 , ^###^*P*<0.0 0 1 *vs*. Normal group ; ^&^*P*<0.05 , ^&&^*P*<0.01 *vs*. LPS+PHE/ ATL-1 group.

**Fig. (4):**
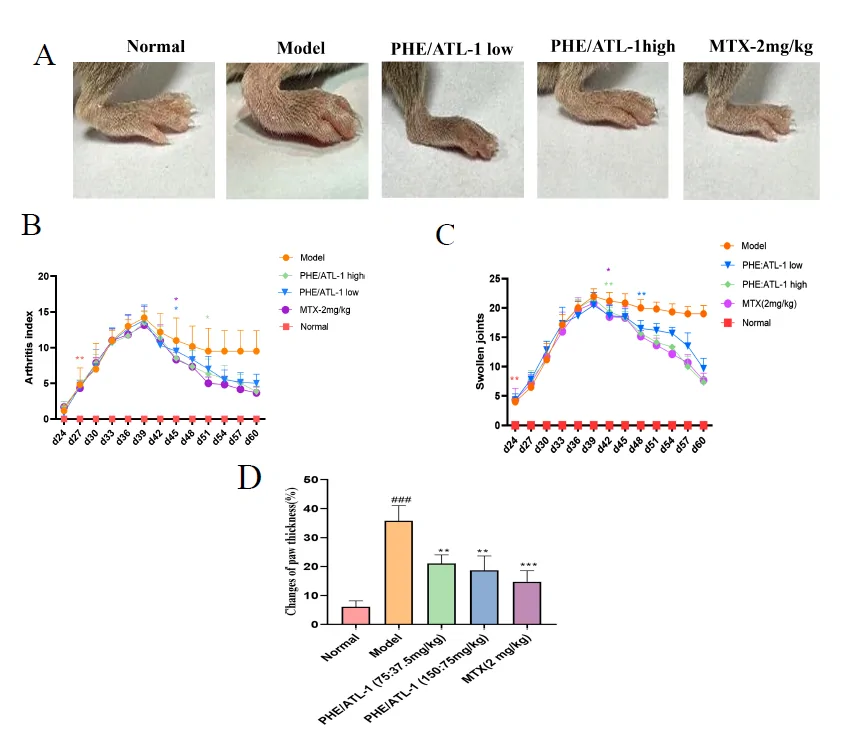
PHE/ATL-1 alleviates arthritis symptoms in CIA mice. (**A**) Representative graph of paw swelling inDBA/1 mice. (**B**) Arthritis in dex. (**C**) Swollen joint count. (**D**) Growth rate of paw thickness. *P<0.05,**P<0.01,***P<0.001*vs*. Model group;^###^*P*<0.001 *vs*. Normal group.

**Fig. (5):**
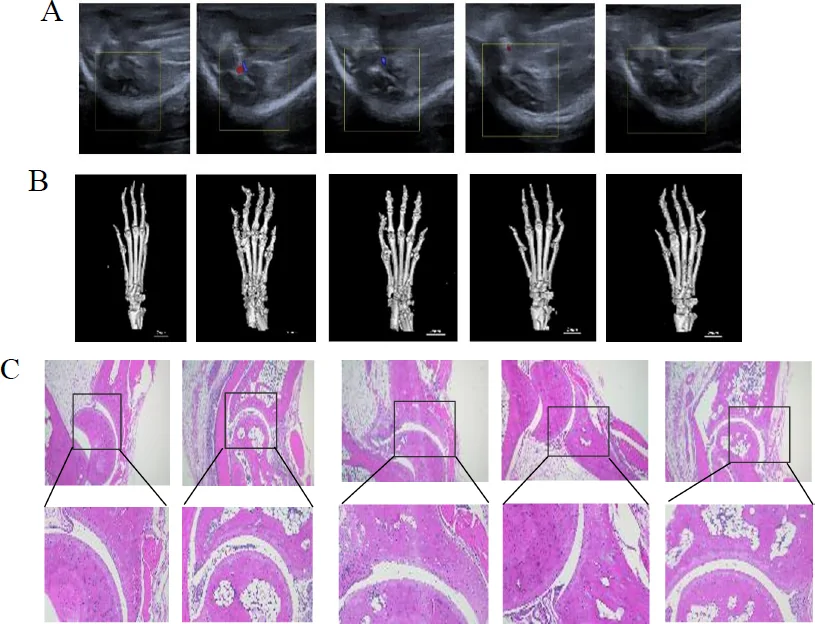
Histopathology of PHE/ATL-1 treatment of arthritis symptoms in CIA mice. (**A**) Knee blood flow signal in mice. (**B**) Micro-CT in mice. (**C**) H & E staining of the ankle joint (H & E ×10 magnification).

**Fig. (6):**
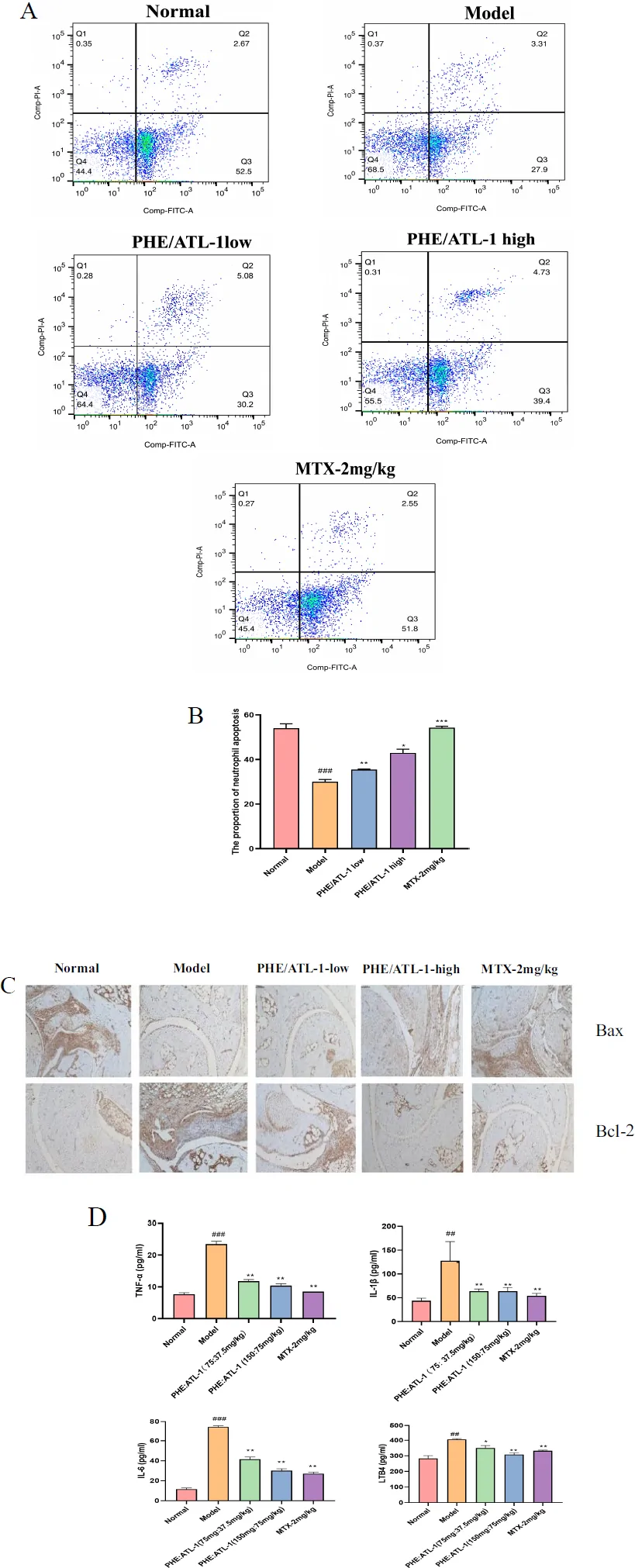
PPHE/ATL-1 promotes neutrophil apoptosis and inhibits inflammation in mice. (**A** and **B**) Flow cytometrywas used to detect the rate of apoptosis. (**C**) Immunohistochemistry was used to detect apoptosis. (**D**) Expression levels of TNF-α, IL-6, IL-1β, LTB4 in the serum of CIA mice. *P<0.05, **P<0.01, ***P<0.001*vs.* Model group;^##^*P*<0.01,^###^*P*<0.001*vs.* ModelNormalgroup. (x̄ x̄ ±s,n=6).

**Table 1:** Basic demographic informationof blood samples collected from 30 rheumatoid arthritis patients in the therapeutic evaluation of PHE/ATL-1.

**No.**	**Gender**	**Age**
RA1	FEMALE	41
RA2	FEMALE	70
RA3	FEMALE	58
RA4	FEMALE	17
RA5	FEMALE	47
RA6	FEMALE	21
RA7	FEMALE	55
RA8	MALE	72
RA9	FEMALE	66
RA10	FEMALE	68
RA11	FEMALE	59
RA12	FEMALE	64
RA13	FEMALE	55
RA14	FEMALE	62
RA15	FEMALE	55
RA16	FEMALE	55
RA17	FEMALE	52
RA18	MALE	60
RA19	MALE	57
RA20	MALE	67
RA21	FEMALE	51
RA22	FEMALE	58
RA23	FEMALE	58
RA24	FEMALE	35
RA25	MALE	71
RA26	MALE	75
RA27	FEMALE	53
RA28	MALE	79
RA29	FEMALE	51
RA30	FEMALE	58

## Data Availability

All data generated or analyzed during this study are included within the article.

## LIST OF ABBREVIATIONS

PHE: Phellodendrine
ATL-1: Atractylenolide-I
RA: Rheumatoid Arthritis
LTB4: Leukotriene B4
BLT1: Leukotriene B4 Receptor 1
CIA: Collagen-induced Arthritis
HE: Hematoxylin-eosin Staining
Micro-CT: Micro-computed Tomography
WB: Western Blot
